# Clinical Spectrum, Molecular Characterization, Antifungal Susceptibility Testing of *Exophiala* spp. From India and Description of a Novel *Exophiala* Species*, E. arunalokei* sp. nov

**DOI:** 10.3389/fcimb.2021.686120

**Published:** 2021-07-02

**Authors:** Shreya Singh, Shivaprakash M. Rudramurthy, Arvind A. Padhye, Basavaraj M. Hemashetter, Ranganathan Iyer, Vinaykumar Hallur, Anuradha Sharma, Sourav Agnihotri, Sunita Gupta, Anup Ghosh, Harsimran Kaur

**Affiliations:** ^1^ Department of Medical Microbiology, Post Graduate Institute of Medical Education and Research (PGIMER), Chandigarh, India; ^2^ Retired, Atlanta, GA, United States; ^3^ Department of Microbiology, Hitech Laboratories, Belagavi, India; ^4^ Department of Microbiology and Infectious Diseases, Global Hospitals, Hyderabad, India; ^5^ Department of Microbiology, All India Institute of Medical Sciences, Bhubaneswar, India; ^6^ Department of Microbiology, All India Institute of Medical Sciences, Jodhpur, India

**Keywords:** *Exophiala*, India, molecular, novel species, antifungal susceptibility

## Abstract

**Introduction:**

*Exophiala* spp. are important opportunist pathogens causing subcutaneous or even fatal disseminated infections in otherwise both immunosuppressed and healthy individuals but there are no systematic studies on the isolates of *Exophiala* species from India.

**Methods:**

Twenty-four isolates of *Exophiala* species were retrieved from the National Culture Collection of Pathogenic Fungi (NCCPF) and identified phenotypically and by molecular methods (ITS region sequencing) followed by antifungal susceptibility testing (AFST) as per CLSI-M38A3 guidelines. A review of the literature of cases from India was performed up to 1^st^ January 2021 using the Medline and Cochrane database.

**Results:**

*E. dermatitidis* (n = 8), *E. jeanselmei* (n = 6), *E. spinifera* (n = 6), *E. mesophila* (n = 1), *E. oligosperma* (n = 1), *E. xenobiotica* (n = 1) were identified and the sequencing of *ITS, β-tubulin* and *β-actin* revealed a novel species, *E. arunalokei* sp. nov. (n = 1). The ITS sequence phylogram of *E. jeanselmei* revealed that the majority (83%) formed a separate cluster close to type A while majority (75%) of *E. dermatitidis* were type B. The MIC50 (mg/L) of amphotericin, itraconazole, voriconazole, micafungin, caspofungin, anidulafungin, and posaconazole, was 1, 0.25, 0.125, 0.12, 0.125, 0.062, and 0.062, respectively. Sixteen more cases were identified on the literature review and a significant association of *E. dermatitidis* with history of surgical procedures (p = 0.013), invasive disease (p = 0.032) and of *E. mesophila* with tuberculosis (p = 0.026) was seen.

**Conclusion:**

This, to the best of our knowledge is the first study from India elucidating the molecular and clinical characteristics of *Exophiala* species and the first Indian report of human infection due to *E. xenobiotica* and *E. arunalokei.*

## Introduction

*Exophiala* species are dematiaceous (black-pigmented) saprophytic molds that are frequently isolated from hot and humid environments that are nutrient deficient but rich in hydrocarbons (Lian and De Hoog, 2010; Zhao et al., 2010; Woo et al., 2013). This genus is a member of the family *Herpotrichiellaceae* and encompasses >30 species. They are often referred to as black yeasts due to the early phenotypic character of their colonies (Matsumoto et al., 1987; Woo et al., 2013). Apart from isolation from environmental samples, they also colonize the human intestine and respiratory tract particularly in the airway of patients with cystic fibrosis (De Hoog et al., 2005; Kondori et al., 2011). However, they must not be disregarded as mere commensals or contaminants as they are also important opportunist pathogens causing systemic infections in immunosuppressed patients and subcutaneous or even fatal disseminated infections in otherwise healthy individuals (Fothergill, 1996; Zeng et al., 2007). Although *Exophiala* species are relatively uncommon, various reports of this genus as an etiologic agent of phaeohyphomycosis are being described from India in recent years (Sood et al., 2014; Venkateshwar et al., 2014; Bhardwaj et al., 2016; Haridasan et al., 2017). Despite the apparent virulence of these species and worrisome clinical scenario, there are no systematic studies on the Indian isolates of *Exophiala* species. Additionally, there is sparse information on the susceptibility profile of Indian isolates of *Exophiala* species against the commonly available antifunga agents. In this study, we aimed to describe the clinical spectrum of infections due to *Exophiala* from India along with phenotypic and molecular characterization and antifungal susceptibility of *Exophiala* species. We also describe a potentially novel *Exophiala* species from a case of subcutaneous phaeohyphomycosis. The novel species exhibited distinct phenotypic characteristics and three independent DNA regions were sequenced to show that it is distinct compared to all other *Exophiala* species. Based on these findings, we propose a new species, *Exophiala arunalokei* sp. nov.

## Material and Methods

### Ethics

The study was approved by the Institute ethical committee of Post Graduate Institute of Medical Education and Research (PGIMER), Chandigarh. Since all clinical data were retrospectively collected from patient records informed consent was waived.

### Patients and Strains

The isolates of *Exophiala* species included in this study were retrieved from the National Culture Collection of Pathogenic Fungi (NCCPF), Chandigarh, India. These isolates were received from various parts of the country during a period of 12 years (2008 through 2020) and clinical data were collected from patient records and NCCPF requisition forms. The identification of all strains was confirmed by molecular methods as described below.

### Conventional Identification

Phenotypic characterization was performed by subculture on malt extract agar (MEA, HiMedia) at 30°C for 7 days followed by subculture on Sabouraud’s dextrose agar (SDA, HiMedia) incubated at 25°C. Colony characters such as texture, color, and rate of growth were recorded. Microscopic features were studied by preparation of the lactophenol cotton blue (LCB) from slide culture (De Hoog et al., 2000). Also, the culture of *Exophiala arunalokei* sp. nov. was inoculated on potato dextrose agar and Oatmeal agar and incubated at 28, 37, and 45°C for up to 2 weeks to study temperature tolerance and to observe a difference in growth characteristics on different media.

### Scanning Electron Microscopy (SEM)

The SEM was performed for one isolate each of *E. jeanselmei*, *E. oligosperma*, and *E. arunalokei* sp. nov. The coverslips from slide culture were used for SEM as previously described (Alves et al., 2013). Briefly, the culture growth over the coverslip was fixed using 2.5% glutaraldehyde followed by gentle washing with phosphate-buffered saline. Then gradient dehydration was performed using different concentration of ethanol (60, 70, 80% and absolute ethanol) followed by air-drying of the sample. The coverslip was loaded onto SEM stub with carbon tape and sputter-coated with platinum at 20 mA current for 30 s and placed onto the SEM stage for reading using the scanning electron microscope JSM I T300 InTouchScope (Jeol, Tokyo, Japan) with low vacuum mode (pressure range from 10 to 650 Pa). All samples were screened at ×250 magnification to identify the area of growth followed by subsequent visualization at ×1,000, ×2,500, and ×5,000 magnification.

### Molecular Identification

For DNA extraction, freshly grown culture growth was transferred to mortar and pestle followed by treatment with liquid nitrogen. Crushed mycelia were suspended in 500 µl of lysis buffer followed by incubation at 56°C for 5 min. DNA was then extracted by the phenol-chloroform-isoamyl alcohol (25:24:1) (PCI) method as per the methods described previously (Shivaprakash et al., 2011). Briefly, the PCI mixture (Sigma Aldrich Inc., MO, USA) was added and mixed well with the lysed specimen followed by centrifugation at 13,000 rpm for 15 min. Then we transferred the aqueous phase to a sterile microcentrifuge tube and DNA was precipitated with absolute ethanol at −20°C overnight. DNA was then centrifuged followed by 70% ethanol wash and then air-dried. It was suspended by thoroughly mixing in 50 µl of sterile, deionized water (Milli Q, Millipore systems) and the yield and quality of the DNA were assessed by spectrophotometric measurement at a wavelength of 260 nm (NanoDrop 2000, Thermo Scientific, Wilmington, DE, USA). Molecular identification was done by sequencing the internal transcribed spacer (ITS) region of ribosomal DNA as described previously (White et al., 1990). The potentially novel *Exophiala* spp was confirmed by sequencing of the β-tubulin gene and β-actin gene using previously published primers (Woo et al., 2013).

### Phylogenetic Characterization

For identification, the ITS sequences of all isolates were aligned with reference sequences at both the online sequence alignment portals, GenBank and International Society of Human and Animal Mycology (ISHAM)-ITS reference DNA barcoding database. The sequences and their relative strains representing each *Exophiala* species obtained from GenBank were used for alignment by the Molecular Evolutionarily Genetics Analysis (MEGA) Version 7.0 software (Kumar et al., 2016). A phylogenetic tree was constructed by neighbor joining (NJ) analysis and the evolutionary relationship among individual isolates was described as horizontal branches. The GenBank accession numbers of all the ITS gene sequences of included *Exophiala* strains are listed in **Supplementary Table S1**.

### Matrix Assisted Laser Desorption–Time of Flight (MALDI-TOF)

The fresh growth of representative isolates belonging to each sequence confirmed species of *Exophiala* were used for off plate protein extraction using formic acid as per published protocol (Paul et al., 2018). For the MALDI-TOF analysis, six isolates were included (five clinical isolates and one reference isolate of *Exophiala spinifera*, ID B2762 CDC, Atlanta). *E. mesophiala* isolate could not be revived and was therefore not included in the MALDI-TOF analysis. MALDI-TOF identification was carried out on MALDI Microflex LT mass spectrometer (Bruker Daltonik GmbH, Bremen, Germany). The spectra of the isolates were added to the Bruker MALDI Biotyper database as described previously (Paul et al., 2018). Briefly, ionized spectral mass ranging from 2 to 20 kDa were acquired at a laser frequency of 60 Hz and MALDI BioTyper v3.1 was used for automated smoothing, normalization, baseline subtraction, and peak selection of different spectra. MALDI-TOF MS-based typing was performed using principal component analysis (PCA) for common species and a minimum of three MSPs (main spectrum profile) were used to construct a dendrogram using MALDI Biotyper software (MALDI Biotyper 3.1, Bruker Daltonics, MC, Italy) after smoothening, baseline subtraction, and normalization of spectra.

### Antifungal Susceptibility Testing

Antifungal susceptibility testing was performed for amphotericin B, fluconazole, itraconazole, voriconazole, posaconazole, isavuconazole, caspofungin, and anidulafungin as per M38A3 protocol of Clinical Laboratory Standards Institute (CLSI, 2017). Briefly, the antifungals were diluted in the standard RPMI 1640 medium and dispensed into 96-well microdilution plates. The final concentration was 0.016–16 μg/ml for amphotericin-B, voriconazole, itraconazole, posaconazole, isavuconazole, and caspofungin; 0.08–8 μg/ml for anidulafungin and 0.063–64 μg/ml for fluconazole. Inoculum suspension in sterile saline with tween 40 was prepared from all isolates grown on SDA plates for 7 days. They were transferred to sterile tubes and optical density (OD) at 530 nm was adjusted to range from 0.09 to 0.13. The final suspension of the isolates ranged from 0.4 × 10^4^ to 3.1 × 10^4^ as confirmed hemocytometer counting and was diluted (1:50) in RPMI 1640 for final use. Microtiter plates were incubated at 35°C and the minimum inhibitory concentration (MIC) endpoints were noted (CLSI, 2017).

### Literature Review

We conducted an electronic search on Medline and Cochrane Library for studies in the English language published up to 1^st^ January 2021. The search was based on the Mesh terms for the keywords (*Exophiala*) AND (India). Only articles reporting human infections were included. We included case reports, brief communications, letter to the editor, and published abstracts. The list of references in the original articles and reviews were searched manually searched to include any additional studies missed by the electronic search. The data extraction was performed using predesigned excel data extraction sheet and the author’s name, year of publication, state/region of study, patient’s clinical and demographic details, mycological findings, treatment, and clinical outcome was documented.

## Results

**Figure 1:**
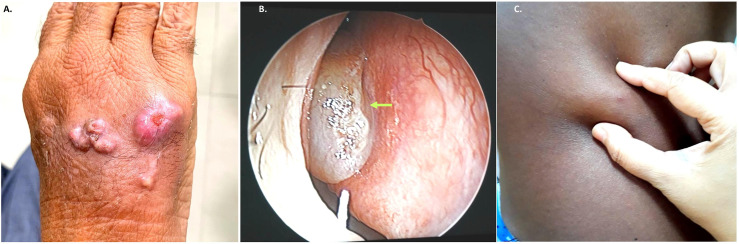
Representative clinical images from subcutaneous infection due to *Exophiala* species. **(A)**. Case no. NCCPF 106025 multiple subcutaneous nodular lesions over dorsum of left-hand **(B)**. Case no. NCCPF 106030 nasal polyps (arrow) in patient with allergic fungal rhino sinusitis **(C)**. Case no. NCCPF 106028 single subcutaneous cystic swelling skin over chest (left side).

A total of 24 isolates of *Exophiala* species from 23 patients could be revived from our culture repository. This included *E. dermatitidis* (n = 8 isolates), *E. jeanselmei* (n = 7 isolates), *E. spinifera* (n = 5 isolates), *E. mesophila* (n = 1 isolate)*, E. oligosperma* (n = 1 isolate), *E. xenobiotica* (n = 1 isolate), and *E. arunalokei* sp. nov. (n = 1 isolate). The clinical profile and microbiological findings of cases are shown in **Supplementary Table S1** and **Figure 1**.

### Phenotypic Identification

**Figure 2:**
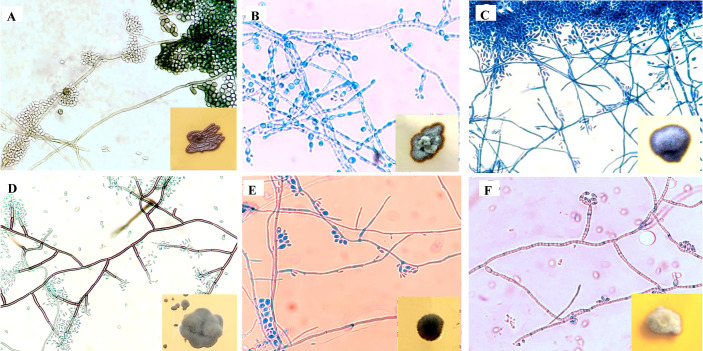
Microscopic findings on lactophenol cotton blue mount from slide culture and gross morphology on obverse surface of Sabouraud’s dextrose agar after 3–7 days at 25°C (inset). **(A)** *E. dermatitidis*, **(B)** *E. mesophile*, **(C)** *E. jeanselmei*, **(D)** *E. spinifera*, **(E)** *E. xenobiotica*, **(F)** *E. mesophila*.

The gross and microscopic morphological characteristics of the representative isolates from various *Exophiala* species are shown in **Figure 2**. Salient identification features of *Exophiala* species prevalent in India is provided in **Table 1**.

**Table 1:** Macroscopic and microscopic details of various *Exophiala* species characterized in the present study.

Exophiala species	Gross morphology (Growth on Sabouraud’s dextrose agar at 25°C)			Microscopy Findings (Lactophenol cotton blue mount from slide culture)		
	Growth rate (size at 7 days)	Obverse surface	Reverse surface	Yeast forms	Annellophore	Conidiogenous cell and annelloconidia
***E. dermatitidis***	5–7 mm	Moist yeast like brown colored initially and developed grayish aerial mycelia on the periphery on day 10–12.	Brownish black	Predominance of pigmented oval to round, budding yeast cells 3–5.2 × 2.2–4.5 µm in size, were seen	The annellophore were lageniform to obclavate, unbranched, 7–8 × 1–3 µm in size with polyblastic conidiogenous cell.	Oval, 1–3 × 2–3 µm in size, mostly single celled (some two celled). Secondary conidia (oval, 1–3 × 2–3 µm in size) arising laterally from the hyphae and agglomerating in clusters around it were seen.
***E. jeanselmei***	3–5 mm	Initially moist later turning flocculose with development of aerial grayish white mycelial growth by 5–7 days which became olivaceous gray over time	Olivaceous black	Few ovoidal yeast cells, 5–10 × 2.4–5.53 µm in size with short pseudohyphae (three to five celled) with more hyaline hyphae	Annellophores were seen, cylindrical to obclavate, 5–12 × 2–3 µm in size.	Both monoblastic (predominant) and polyblastic conidiogenous cell were present. Annelloconidia were hyaline single celled, oval, 2–4 × 1–2 µm in size.
***E. spinifera***	4–6 mm	Initially moist shiny and black, becoming pulvinate to velvety and olivaceous gray	Olivaceous black	Few oval yeast cells (2–4 × 3–5 µm**)** with more subhyaline to brown colored hyphae	Pigmented, infrequently branched, cylindrical, lageniform, obclavate to cylindrical in shape, approximately 16.4–26.2 × 1–3 µm in size. Darkened spine like prominence was seen terminally on annellophore.	Conidiogenous cells were predominantly polyblastic and had snout like annellated apices. Conidia were subhyaline, single celled, oval to clavate, 2–4 × 1–2 µm in size. Secondary conidia arising from hyphae were also seen but less frequently than *E. jeanselmei.*
***E. xenobiotica***	3–6 mm	Moist yeast like brown colored initially developed grey velvety mycelia at periphery	Black to gray	Few pigmented oval to round yeast cells were seen (3–5 × 2–4 µm in size) with sub-hyaline hyphae (3 µm diameter).	Few, short, obclavate, branched, 3–5 × 1–3 µm in size	Polyblastic conidiogenous cell were seen. Sessile, oval conidia were more frequent (1–3 × 2–3 µm in size)
***E. oligosperma***	5–7 mm	Olivaceous, brown velvety growth	Black	Yeast cells were 7.6–13.06 × 14.7–19 µm in size.	Pale brown hyphae were seen and annellophore were long (6–10.3 × 12.1–19.1 µm), branched	Sparse conidia (1–3 × 2–3 µm) from both intercalary loci along the hyphae (3 µm diameter).
***E. mesophila***	6–8 mm	Black and yeast like initially, with grayish black and velvety filamentous areas in few days	Black	Few yeast cells with annellidic protrusions were seen (4–5 × 5–7 µm in size)	Obclavate to cylindrical in shape, approximately 1.4–6.5 × 1–3 µm in size.	The annelids were tubular and 9–12 µm in length and conidia were seen terminally as well as at intercalary loci.
***E. arunalokei* sp. nov.**	2–4 mm	Yeast like initially, with greenish- gray and velvety filamentous growth in 2–3 days	Black	Few ovoidal, hyaline yeast-like cells: 4–6 × 2–3.6 µm in size.	Vegetative hyphae were subhyaline, olive to black brown, branched, and septate (1–2.5 µm in diameter). Cylindrical annellophores (6–11.2 × 1–3.2 µm)	Predominantly polyblastic oval secondary conidia were seen terminally with a slight prominence at tip (less evident than seen in *E. spinifera*) or laterally from hyphae. Annelloconidia were hyaline single celled, oval, 2–4 × 1–2 µm in size.

### *E. arunalokei* sp. nov.

**Figure 3:**
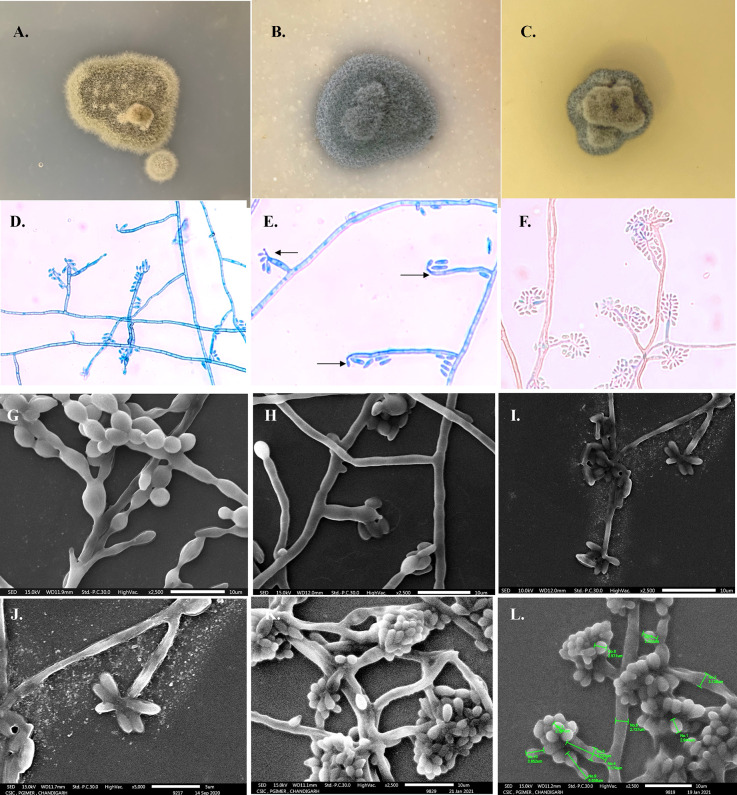
Phenotypic characteristics of *E arunalokei* sp. nov. Gross morphology on obverse surface culture growth after 14 days at 28°C on **(A)** Potato dextrose agar, **(B)** Oatmeal agar, and **(C)** Sabouraud’s dextrose agar. **(D)** Microscopic findings from slide culture (day 10) on lactophenol cotton blue mount showing branched annellophore, **(E)** terminal prominence (arrow), **(F)** LCB from slide culture on day 21. Scanning electron microscopy (SEM) of **(G)** *E oligosperma*, **(H)** *E. jeanselmei*, and **(I)** *E. arunalokei* sp. nov. day 10, ×2,500; **(J)** ×5,000 (day 10); **(K)** day 21, ×2,500, **(L)** day 21, ×2,500 with size measurements.

Growth on SDA was black and yeast-like initially, after 7 days of incubation at 25°C filamentous growth appearing greenish-gray and velvety of ~2 mm in diameter was noted with black pigmentation on reverse surface. Microscopic examination of the slide culture revealed hyaline hyphae and few hyaline yeast-like cells: ovoidal, 4–6 × 2–3.6 µm in size. Vegetative hyphae were subhyaline, olive to black-brown, branched, and septate (1–2.5 µm in diameter). Cylindrical annellophores (6–11.2 × 1–3.2 µm) with many predominantly polyblastic oval secondary conidia were seen terminally with a slight prominence at the tip (less evident than seen in *E. spinifera*) or laterally from hyphae. Annelloconidia were hyaline single-celled, oval, 2–4 × 1–2 µm in size. Even on SEM, the size of the conidiophores was 7.1–11.36 × 2.0–2.7 µm and the conidia ranged from 2.2–3.93 × 1.5–2.09 µm. **Figure 3** shows the phenotypic characteristics of *E. arunalokei* sp. nov. as seen on culture, light microscopy, and scanning electron microscopy. *E. arunalokei* sp. nov. grew rapidly on PDA and OMA compared to SDA agar. Optimal growth of *E. arunalokei* sp. nov. was seen at 28°C, with less growth at 37°C and no growth at 45°C.

**Figure 4:**
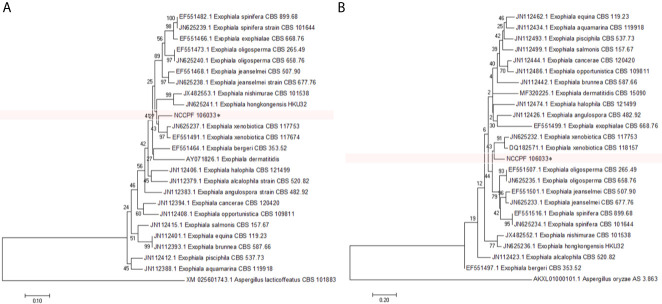
Phylogenetic tree showing the relationship of *E. arunalokei* to closely related species using **(A)** β-actin gene and **(B)** β-tubulin gene sequencing data by using the Neighbor-Joining method clustered together in the bootstrap test (1,000 replicates); conducted in MEGA 7.

The amplicon size of the β-tubulin and β-actin genes of *E. arunalokei* sp. nov. and the reference strains were ~300 and ~500 bp, respectively. *E. arunalokei* sp. nov. was found to be most closely related to *E. xenobiotica* [90% similarity with β-actin gene (JN625237.1) and 80% similarity with β-tubulin gene (JN625236.1)] on phylogenetic analysis (**Figure 4**) concurrent with the findings of ITS gene sequencing (91% similarity with *E. xenobiotica* CBS204.50, KP132163).

*Taxonomy of Exophiala arunalokei* sp. nov*.: Exophiala arunalokei* Singh and Rudramurthy sp. nov. MycoBank accession no. is awaited. Teleomorph: unknown. Known distribution: India. Etymology: a.ru.na.lo’ke.i. N.L. masc. gen. n. arunalokei, of Arunalokei in honor of Professor Arunaloke Chakrabarti, for his many academic and scientific contributions in the field of medical mycology. Specimen examined: in India; from a subcutaneous lesion on the face of a human presenting with subcutaneous phaeohyphomycosis in 2020. Based on the microscopic morphological features and sequence analysis of the ITS region, beta-actin and beta-tubulin region presented above, the isolate is proposed as the strain of a novel species in the genus *Exophiala*. Holotype: dried culture at the NCCPF, Chandigarh, India (*E. arunalokei* NCCPF 106033).

### PCR and Sequence Analysis

**Figure 5:**
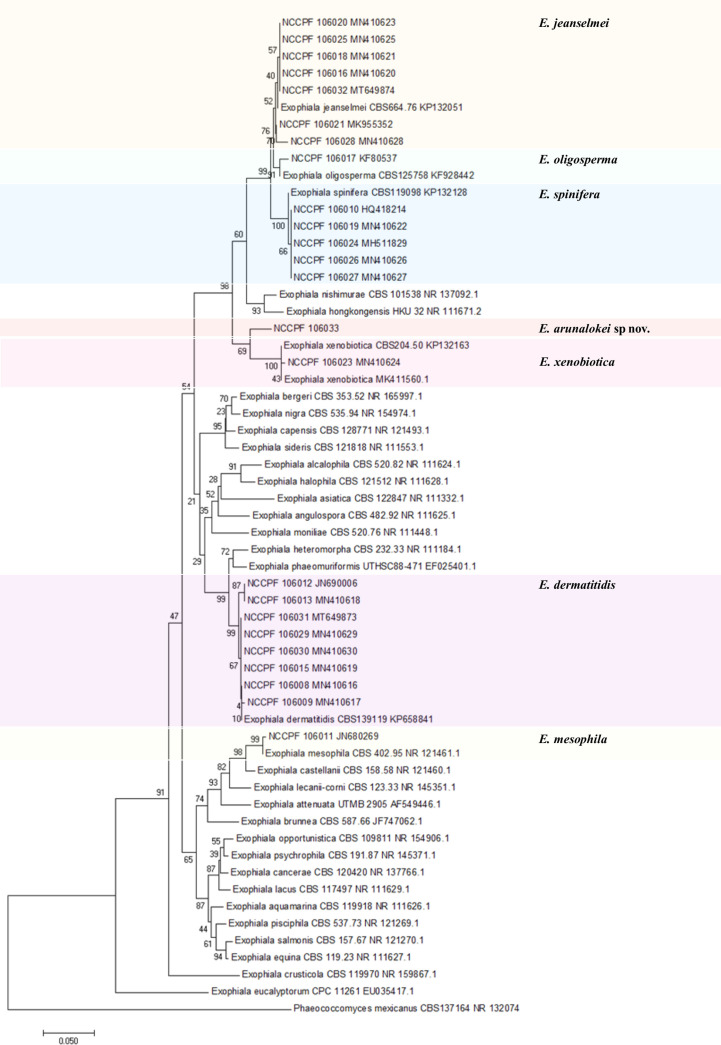
Evolutionary relationships of 24 isolates of *Exophialia* species with Neighbor-joining algorithm with 1,000 bootstrap replicates. *Phaeococcomyces mexicanus* CBS 127164 is taken as out-group. ITS, internal transcribed spacer.

The ITS sequences of the 24 Indian isolates of *Exophiala* species recovered from our collection measures ~600 bp. **Figure 5** depicts the phylogram prepared from the ITS sequences of all 24 clinical isolates of the present study and 34 strains from various species of *Exophiala* reported from clinical and environmental samples. The aligned sequences of various previously published type strains *E. jeanselmei* (Types A, B, and C) and *E. dermatitidis* (Types A0, A1, A2, and B) were also used to study the evolutionary relationship of *E. jeanselmei* and *E. dermatitidis* isolates in our study with previously described type isolates is shown in **Supplementary Figures 1** and **2** (Suh et al., 2012). Two isolates of *E. jeanselmei* (NCCPF 106028 and 106021) from cases of subcutaneous infection showed sequence similarity with *E. jeanselmei* Type B while the remaining five isolates clustered separately but close to *E. jeanselmei* Type A, suggesting the possibility of a distinct type or subtype. The phylogram of ITS sequences of *E. dermatitidis* isolates showed that majority of the isolates clustered with *E. dermatitidis* Type B. However, the isolates number NCCPF 106012 and NCCPF 106013 were both recovered from the same patient with endocarditis, and they clustered with *E. dermatitidis* type A isolates.

### MALDI-TOF Analysis

None of the six isolates used in initial evaluation could be correctly identified using the existing Bruker database. A total of 24 spectra for each isolate were subjected to smoothing, normalization, baseline subtraction and added to our in-house database to expand the spectral library. The novel species *E. arunalokei* sp. nov., had a distinct spectral profile which was most similar to *E. jeanselmei* (**Supplementary Figure 3A**). Subsequently, *E. dermatitidis* (n = 7), *E. jeanselmei* (n = 5), *E. spinifera* (n = 6), and one isolate each of *E. oligosperma*, *E. xenobiotica*, and *E. arunalokei* sp. nov., were identified using the expanded in-house database and all could be correctly identified at a log score value of ≥1.7. Since only one isolate each of *E. oligosperma*, *E. xenobiotica*, and *E. arunalokei* sp. nov. were available the same isolate was used during in-house spectral database expansion and validation. The PCA dendrogram obtained by MALDI-TOF MS of the common *Exophiala* species revealed clusters which concurred with the sequencing results and dendrogram with representative species is shown in **Supplementary Figure 3B**.

### Antifungal Susceptibility Testing (AFST)

A total of 24 isolates were subjected to AFST and the MIC (mg/L) ranged from 0.03 to 4 for amphotericin, 0.03 to 2 for all tested triazoles (itraconazole, voriconazole, and posaconazole), 0.125 to 16 for micafungin and caspofungin, and 0.03 to 16 for anidulafungin. The MIC of echinocandins was >4 mg/L in 62.5% (n = 5) isolates of *E. dermatitidis* and in one (16.7%) isolate of *E. spinifera*. The MIC of voriconazole was high (>2 mg/L) in an isolate each of *E. oligosperma* and *E. mesophila* (**Table 2**). The isolate of *E. arunalokei* sp. nov., had low MICs for all antifungals tested with a high MIC of 4 mg/L for amphotericin B.

**Table 2:** Antifungal susceptibility testing of clinical isolates of *Exophiala* species.

	AMB	ITC	VRC	POS	MFG*	CAS	AFG
**Total (n = 24)**							
GM	0.655	0.141	0.891	0.116	0.098	0.5	0.564
Range	0.03–4	0.03–2	0.03–2	0.03–0.5	0.125–16	0.125–16	0.03–16
MIC50	1	0.25	0.12	0.062	0.125	0.125	0.062
***E. dermatitidis* (n = 8)**							
GM	0.707	0.177	3.482	0.134	0.162	1.542	1.834
Range	0.25–2	0.03–0.5	0.03–2	0.03–0.5	0.125–16	0.125–16	0.03–8
MIC50	0.5	0.5	0.062	0.25	4	4	4
***E. jeanselmei* (n = 7)**							
GM	0.660	0.054	0.141	0.072	0.125	0.287	0.094
Range	0.2–4	0.03–0.25	0.06–0.25	0.03–0.25	0.125	0.125–2	0.03–2
MIC50	0.5	0.031	0.12	0.031	0.125	0.125	0.0312
***E. spinifera* (n = 5)**							
GM	1	0.353	0.125	0.097	0.099	0.561	0.445
Range	0.25–2	0.03–1.0	0.03–0.25	0.03–0.5	0.125–2	0.03–4	0.03–16
MIC50	1	0.5	0.062	0.031	0.5	0.125	0.031
***E. oligosperma* (n = 1)**	0.031	0.031	4	2	0.031	0.125	0.125
***E. mesophila* (n = 1)**	0.25	0.062	2	0.031	0.031	0.125	0.125
***E. xenobiotica* (n = 1)**	0.25	0.062	0.125	0.031	0.031	0.125	0.125
***E. arunalokei* (n = 1)**	4	0.25	0.031	0.25	–	0.5	0.125

*20 isolates tested [E. dermatitidis (n = 8), E. jeanselmei (n = 6), E. spinifera (n = 4), E. mesophila (n = 1), E. xenobiotica (n = 1)].

### Review of Literature and Data Analysis

A total of 27 articles were retrieved after an online and manual literature search. Of these, three were excluded based on title and abstract review. Eight isolates of seven published studies had been submitted at NCCPF and were included in this study as shown previously in **Table 1**. Duplication of publication was seen in two studies and it was counted as a single case (Capoor et al., 2007; Khan et al., 2007). The analysis of case details of 39 cases of *Exophiala* infections [23 included in the present study (**Supplementary Table S1**) and 16 from the literature review (**Supplementary Table S2**)] was performed.

A total of 39 cases due to *Exophiala* species have been reported from India with a median age of 35 years (range 8 to 73 years). The most common species reported was *E. jeanselmei* (n = 16, 41.02%), followed by *E. dermatitidis* (n = 10, 25.6%), *E. spinifera* (n = 9, 23.1%), *E. oligosperma* (n = 1, 2.6%), *E. mesophila* (n = 1, 2.6%), *E. xenobiotica* (n = 1, 2.6%), and *E. arunalokei* (n = 1, 2.6%). The most common clinical presentation was subcutaneous phaeohyphomycosis (n = 16, 41.02%), followed by invasive/disseminated infection (n = 9, 23.1%), eumycetoma (n = 5, 12.8%), skin infection (n = 3, 7.69%), allergic fungal rhinosinusitis (n = 1, 2.6%), and endophthalmitis (n = 1, 2.6%), while the details of four cases were not available.

The most common risk factor associated with infection (details available in 33) was trauma (n = 11, 33.3%), post-renal transplant immunosuppression (n = 4, 12.1%), history of surgical procedures (n = 3, 9.1%), diabetes (n = 3, 9.1%), malnourishment (n = 2, 6.1%), tuberculosis (n = 1, 3.03%), and chronic hepatitis B (n = 1, 3.03%) while 8 (24.2%) were immunocompetent. In case of infection due to *E. dermatitidis*, a significant association with a history of surgical procedures was seen compared to other species (30 *vs.* 0%; p-value: 0.013). *E. mesophila* infection was significantly associated with a history of tuberculosis compared to other species (100 *vs.* 0%; p-value: 0.026). Analysis of species wise clinical presentation revealed a significant association of *E. dermatitidis* infection with the invasive disease compared to other species (50 *vs.* 10.7%, p-value: 0.032).

The details of patient management were available for 24 cases. Antifungals were used in 19 cases of which the majority received itraconazole (n = 7), followed by voriconazole (n = 4) and ketoconazole (n = 3) while a combination of different antifungals such as amphotericin, voriconazole, itraconazole, fluconazole, and terbinafine was used in six cases. Surgical intervention involving the drainage or debridement of the infected site was performed in 11 and cryotherapy in one case. Outcome details were available for 26 cases of which 24 (92%) recovered while 2 (7.69%) died.

## Discussion

*Exophiala* species are difficult to characterize using phenotypic tests and different molecular methods have been recommended for accurate species identification (Zeng et al., 2007; Woo et al., 2013; Silva et al., 2017). Disagreements while comparing morphological and molecular identification methods have been previously reported for this genus (Zeng et al., 2007). In the present study, we describe the largest Indian casuistic of *Exophiala* spp. isolated from clinical samples and characterized phenotypically and by ITS sequencing for accurate species-level identification. Since molecular characterization of isolates is not always possible at hospital based laboratories we also performed the MALDI-TOF MS base identification of our isolates and none of them could be correctly identified up to species level using the existing Bruker database. Hence, we expanded our in-house library of protein spectra.

The clinical details of cases included in the present study and the data recovered by a systematic review of previous Indian studies were analyzed to elaborate on the spectrum of clinical *Exophiala* species from India. A large diversity of species was demonstrated, with *E. jeanselmei* being the most prevalent followed by *E. dermatitidis*. This is also the first Indian study, to report a case of human infection due to *E. xenobiotica.* Phylogenetic analysis of sequencing data from three independent regions i.e., ITS and two housekeeping genes (β-tubulin, and β-actin gene) unambiguously showed that the isolate NCCPF 106033 formed branches distinct from other species and we propose the novel species *E. arunalokei*. This species was related most closely to *E. xenobiotica* evidenced in all phylogenetic trees with high bootstrap support. Compared to *E. jeanselmei*, *E. arunalokei* grew slowly and had smaller conidia with branched conidiophores with a slight prominence at the end. *E. arunalokei* culture growth was velvety compared to *E. xenobiotica* and conidia were also smaller in size.

The sequence typing of all isolates of *E. jeanselmei* and *E. dermatitidis* were performed as reported previously by Suh et al. (Suh et al., 2012) and most Indian isolates of *E. jeanselmei* were found to be related to *E. jeanselmei* type A, while *E. dermatitidis* were Type B. Previous studies analyzing ITS rDNA of *E. dermatitidis* have revealed that type B is commonly constituted by environmental strains while type A isolate are prevalent in the clinical setting (Matos et al., 2003; Suh et al., 2012). Interestingly, while all *E. dermatitidis* isolates in our study caused invasive disease the isolates infecting immunocompetent hosts clustered in type B. We also identified two *E. dermatitidis* Type A2 strains in patients with post-renal transplant immunosuppression. Previously Type A2 has been reported from the sputum of a cystic fibrosis patient from Germany (*E. dermatitidis* CBS 149.90 AF050268). *E. jeanselmei* Type B has been previously reported from subcutaneous infections in humans from Korea and England (Suh et al., 2012). We too found that the two Type B *E. jeanselmei* isolates in our study, caused subcutaneous infection.

Although clinical breakpoints are not available for *Exophiala* spp. are not available the AFST results show low MICs to all antifungals amongst all the described species, except one isolate each of *E. dermatitidis* and *E. oligosperma* which show high voriconazole MIC. Thus, monitoring the trends of antifungal susceptibility is essential to anticipate the development of antifungal resistance in the future and modify the patient management protocols accordingly. Azoles, especially itraconazole and posaconazole and amphotericin B are the most active antifungals. However, there are no established breakpoints for *Exophiala* species and reports of AFST profile remain sparse (Zeng et al., 2007; Revankar and Sutton, 2010; Silva et al., 2017).

Association of *E. dermatitidis* infection with the invasive disease is concordant with previous reports where this species has been reported as the most virulent in this genus (Revankar and Sutton, 2010; Silva et al., 2017). In case of infection due to *E. dermatitidis*, a significant association with a history of surgical procedures was seen compared to other species (p-value: 0.013) which could indicate a possibility of nosocomial acquisition. Although there was a statistical association between tuberculosis and *E. mesophila* infection (p value: 0.026), since it was only one case, an association at this juncture is debatable. Subclinical airway colonization may be involved in such cases as observed in cases with cystic fibrosis (Kondori et al., 2011). However, no such cases were seen in the present study suggesting the need for prospective studies to assess the prevalence of *Exophiala* spp. in respiratory secretions from such patients in our region. The wide spectrum of infections due to *Exophiala* species in both immunocompetent and immunocompromised patients is evident from this study. In addition to antifungal therapy, concomitant surgical management should be performed whenever possible.

In a study from Hong Kong, characterization from 12 clinical isolates of *Exophiala* spp. revealed that superficial and subcutaneous infections were more common and the most common species recovered was *E. oligosperma* (n = 3; two from cases of onychomycosis and one from case of pneumonia) followed by *E. jeanselmei* (n = 2; both subcutaneous infection), *E. lecanii-corni* (n* =* 2; both subcutaneous infection), *E. bergeri* (*n* = 1; onychomycosis), *E. xenobiotica (*n = 1, peritonitis), and a novel species *E*. *hongkongensis* (case of onychomycosis) (Woo et al., 2013). *Exophiala* strains (n = 185) were characterized in another study from the United States of America, where 50.8% were seen in cutaneous/subcutaneous infection while 39.9% were found to be isolated from deep infections. *E. dermatitidis*, *E. xenobiotica*, and *E. oligosperma* comprised more than two-thirds of the isolates and while systemic infections were mainly caused by *E. dermatitidis*, *E. oligosperma*, and *E. xenobiotica*, *E. spinifera*, *E. jeanselmei*, and *E. mesophila* caused cutaneous/subcutaneous infections (Zeng et al., 2007).

This, to the best of our knowledge, is the first study from India elucidating the molecular and clinical characteristics of *Exophiala* species. We find that the combination of ITS sequence phylogenetic analysis can supplement traditional morphological identification of *Exophiala* species as well as the evaluation of its distribution, determination of types and subtypes. It also enabled us to describe a novel species *E. arunalokei.* Apart from sequence based identification, MALDI-TOF can also serve to be quite useful provided the spectral library is enhanced. We have expanded our in-house database using the spectra acquired from representative isolates in this study however, further expansion and validation of this library is essential for identification with better log scores. The antifungal susceptibility data presented here can also provide an insight into the complete picture of *Exophiala* species from India. This can help in establishment of epidemiological cutoffs for MIC interpretation and should be further investigated in multicenter studies.

## Data Availability Statement

The datasets presented in this study can be found in online repositories. The names of the repository/repositories and accession number(s) can be found in the article/**Supplementary Material**.

## Author Contributions

SS, SR, and AP designed and supervised the study. BH, RI, VH, and AS provided the isolates and performed data collection. SS, SA, and SG carried out the laboratory experiments. SS carried out the data analysis and wrote the manuscript. SR, AG, and HK revised the manuscript. All authors contributed to the article and approved the submitted version.

## Conflict of Interest

Author BH was employed by the company Hitech Laboratories.

The remaining authors declare that the research was conducted in the absence of any commercial or financial relationships that could be construed as a potential conflict of interest.
