# Supplementary material for: Clinical Spectrum, Molecular Characterization, Antifungal Susceptibility Testing of Exophiala spp. From India and Description of a Novel Exophiala Species, E. arunalokei sp. nov
Source: Front Cell Infect Microbiol. 2021 Jul 2;11:686120. doi: 10.3389/fcimb.2021.686120 (PMC8284318; doi:10.3389/fcimb.2021.686120)
Supplement: Supplementary file 2 [file Image_2.pdf]

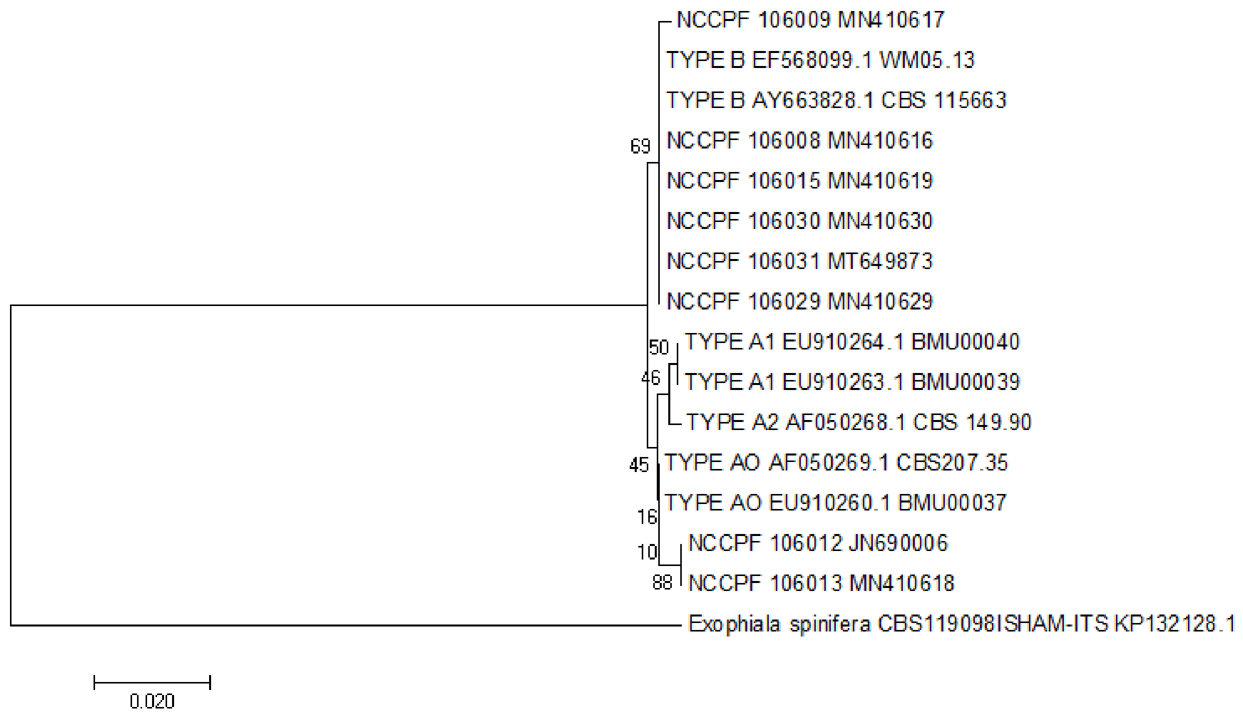

Figure S2. Evolutionary relationships of *E. dermatitidis* using the Neighbor-Joining method clustered together in the bootstrap test (1000 replicates); conducted in MEGA7. *E. spinifera* CBS1190981 used as out-group.
