# Supplementary material for: Clinical Spectrum, Molecular Characterization, Antifungal Susceptibility Testing of Exophiala spp. From India and Description of a Novel Exophiala Species, E. arunalokei sp. nov
Source: Front Cell Infect Microbiol. 2021 Jul 2;11:686120. doi: 10.3389/fcimb.2021.686120 (PMC8284318; doi:10.3389/fcimb.2021.686120)
Supplement: Supplementary file 3 [file Image_3.pdf]

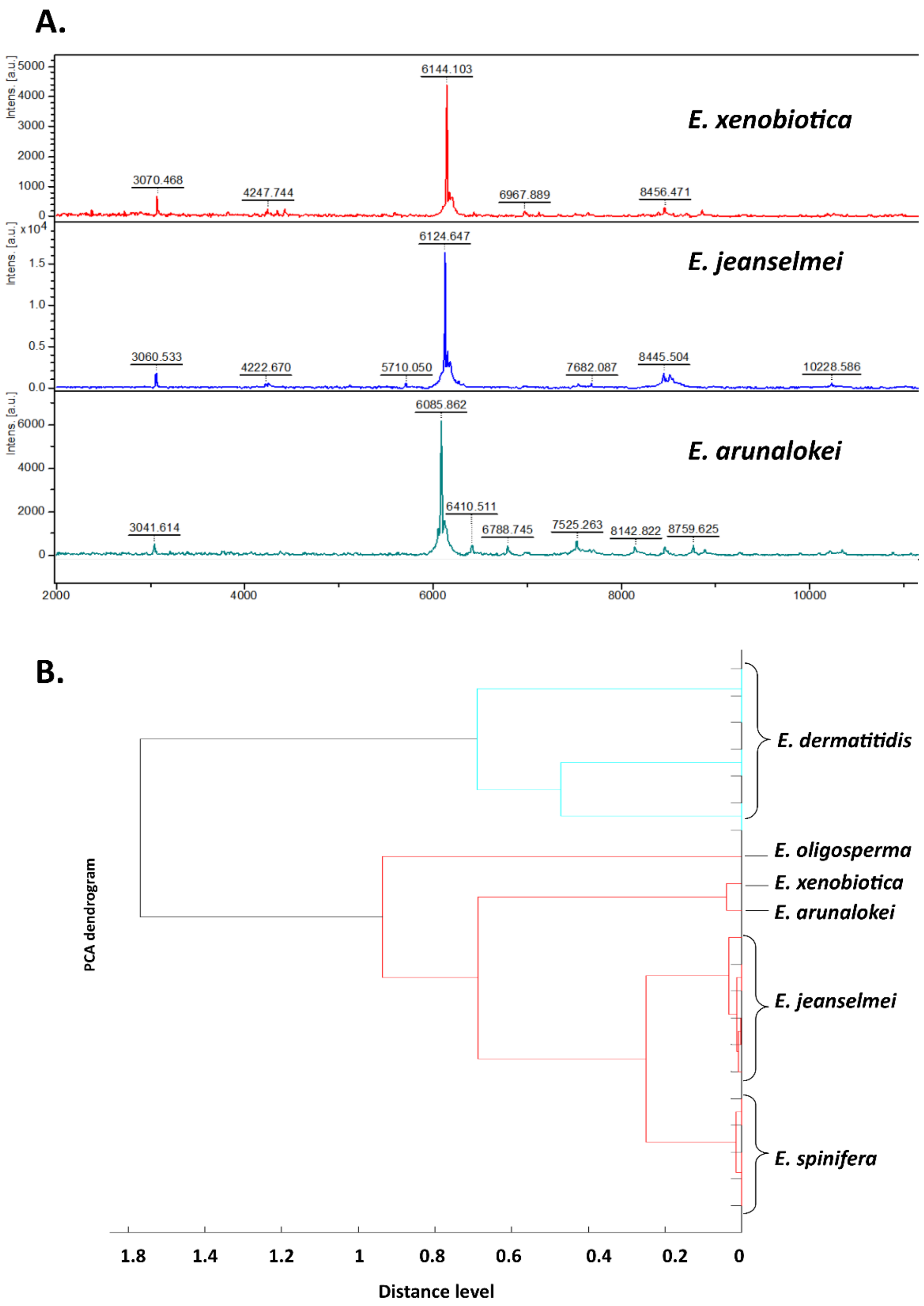

Figure S3. A. Different protein spectral profile of closely related species- *E. xenobiotica*, *E. jeanselmei* and *E. arunalokei B.* Principal component analysis dendrogram depicting the differentiation of common *Exophiala* species by MALDI-TOF MS
