# Supplementary material for: Clinical Spectrum, Molecular Characterization, Antifungal Susceptibility Testing of Exophiala spp. From India and Description of a Novel Exophiala Species, E. arunalokei sp. nov
Source: Front Cell Infect Microbiol. 2021 Jul 2;11:686120. doi: 10.3389/fcimb.2021.686120 (PMC8284318; doi:10.3389/fcimb.2021.686120)
Supplement: Supplementary file 4 [file Table_1.docx]

**Table S1. Clinical and mycological details of the cases of infection due to *Exophiala* spp included in the present study**

NCCPF no. National collection of pathogenic fungi - reference number, M: male, F: female, BD: twice a day, KOH: 10% potassium hydroxide, CT: computed tomography, MRI: magnetic resonance imaging, PAS: periodic acid schiff’s stain, FNAC: fine needle aspiration cytology, GMS: Grocotts methanamine silver stain, H and E: hematoxylin and Eosin stain.

*two isolates recovered from same patient at different sites i.e. blood and aortic valve tissue

| **S.no.** | **Location** | **Year** | **NCCPF-no.** | **Accession no.** | **Species** | **Clinical detail** | **Risk factor** | **Investigation** | **Treatment** | **Outcome** | **Reference** |
| --- | --- | --- | --- | --- | --- | --- | --- | --- | --- | --- | --- |
| 1 | Chandigarh | 2008 | 106008 | MN410616 | *E. dermatitidis* | 22/M, fever and shortness of breath | Patient underwent thoracocentisis and drainage of pleural effusion (1 week before onset) | Chest Xray: Right sided pleural effusion. Pus from pigtail drain: KOH mount and culture positive | Intravenous voriconazole followed by oral voriconazole 200 mg BD | Recovered | Present study |
| 2 | Hyderabad | 2008 | 106009 | MN410617 | *E. dermatitidis* | 35/M, Systemic phaeohyphomycosis with fever and generalized weakness | None | Blood culture positive | Intravenous voriconazole followed by oral voriconazole 200 mg BD | Death | Present study |
| 3 | Chandigarh | 2008 | 106010 | HQ418214 | *E. spinifera* | 26/M laborer pustulonodular lesions with itching and pain over the chin, superior orbital region and nose | None | KOH mount of skin biopsy revealed multiple darkly pigmented hyphae. Culture positive | Itraconazole | Recovered | (Singh et al. 2012) |
| 4 | Assam | 2008 | 106011 | JN680269 | *E. mesophila* | 73/F, severe cough and respiratory distress | Uncontrolled Diabetic(Type II) and Pulmonary tuberculosis | Chest X-ray: left lower lobe consolidation  CT scan thorax: patchy consolidation in superior segment and posteromedial basal segment of the lower lobe of the left lung  Sputum KOH + and culture + | Oral voriconazole 200 mg twice daily | Recovered | (Purnima et al. 2015) |
| 5 | *Gujrat | 2010 | 106012 (Blood)  106013  (aortic valve) | JN690006  MN410618 | *E. dermatitidis* | 50/ F, generalized weakness, dry cough and joint pain in left wrist. On examination diastolic and systolic murmur at aortic area | Post renal transplant (since 4 years) on prednisolone, tacrolimus and mycophenolate | Echocardiography revealed aortic regurgitation with a large,  freely mobile vegetation at the aortic valve | Intravenous voriconazole  and Aortic valve replacement with bioprosthetic valve, Reduction in tacrolimus dose by 1/3rd | Recovered | (Patel et al. 2013) |
| 6 | Jaipur | 2012 | 106015 | MN410619 | *E. dermatitidis* | 21/M, progressive weakness of right hand and slurred speech | Student, history of surgical drainage of abscess in left ear on two occasions in past | MRI  brain: multiple, nodular, conglomerated  lesions in left fronto‑parietal lobes and left capsule‑ganglionic region with surrounding mass effect and oedema Intraoperative tissue histopathology positive for fungal hyphae (PAS satin), KOH and culture positive | Left temporo‑parietal craniotomy and tumour  decompression. Amphotericin  B 3-5 mg/kg/day changed to  Voriconazole 200 mg BD due to renal derangement | Recovered | (Sood et al. 2014) |
| 7 | Kolkatta | 2012 | 106016 | MN410620 | *E. jeanselmei* | 35/M, multiple abscess on both arms | None | Pus aspirate positive on KOH mount and culture | Surgical drain of pus | LFU | Present study |
| 8 | Puducherry | 2014 | 106017 | KF780537 | *E. oligosperma* | 38/M, construction worker  Single non tender nodule, progressively increasing in size at the dorsum of right elbow | Trauma | FNAC: PAS stain positive for hyphae,  KOH +  Culture +, sequencing+ | Surgical excision of the nodule, no antifungal | Recovered | (Venkateshwar et al. 2014) |
| 9 | Lucknow | 2014 | 106018 | MN410621 | *E. jeanselmei* | Subcutaneous phaeohyphomycosis involving the hand and fore arm | None | FNAC pus  KOH + and Culture + | NA | NA | Present study |
| 10 | Bengaluru | 2014 | 106019 | MN410622 | *E. spinifera* | 12/M, slowly progressive, hyperkeratotic, verrucous plaque on left foot and left forearm since 6 months. Progressed to painful swelling of left elbow and weakness in arm and leg(left side) | None | X-ray of left elbow joint expansile osteolytic lesion on the proximal end of left radius. MRI of the brain showed “double ring sign” suggestive of fungal granuloma. Histopathology and KOH mount from skin biopsy was positive for sclerotic bodies | Intravenous voriconazole 6 mg/kg followed by oral voriconazole 100mg BD and itraconazole 100 mg BD for 3 months. Then itraconazole for 6 months | Recovered | (Srinivas et al. 2016) |
| 11 | Chandigarh | 2015 | 106020 | MN410623 | *E. jeanselmei* | 45/M acute onset of sever epigastric abdominal pain | None | CT-Scan revealed enlarged peripancreatic fluid collection and drain fluid was positive on culture | Voriconazole | NA | Present Study |
| 12 | Chandigarh | 2015 | 106021 | MK955352 | *E. jeanselmei* | 52/M , agriculturist with multiple asymptomatic swellings on dorsum of hand and feet. Discharging sinus with yellow discharge | Possible trauma during agricultural activities | Cyst material: Histopathology (PAS and Grocotts) positive and culture positive. | Surgical excision, no antifungal | Recovered | Present Study |
| 13 | Karnataka | 2015 | 106023 | MN410624 | *E. xenobiotica* | 35/M, farmer with subcutaneous swelling and multiple discharging sinuses over dorsum of right foot (mycetoma) | Possible trauma during agricultural activity | FNAC pus from lesion KOH mount and culture positive | Itraconazole | Recovered | Present Study |
| 14 | Chandigarh | 2018 | 106024 | MH511829 | *E. spinifera* | 35/M, Farmer, with itchy, tender, verrucous lesion on face (since 10 years) | None | Histopathology: septate hyphae (PAS, GMS, Masson Fontana stain), KOH mount and culture positive. | Itraconazole | Recovered | (Kapatia et al. 2018) |
| 15 | Chandigarh | 2018 | 106025 | MN410625 | *E.*  *jeanselmei* | 60/ M, single non tender nodule on right hand, progressively increasing in size (2 months) | Renal transplant recipient | FNAC: from swelling, KOH mount and culture positive | Itraconazole | Initial improvement with recurrence. LFU | Present Study |
| 16 | Odisha | 2018 | 106026 | MN410626 | *E. spinifera* | 60/M subcutaneous phaeohyphomycosis | None | FNAC pus from lesion KOH mount and culture positive | Voriconazole | LFU | Present Study |
| 17 | Pune | 2018 | 106027 | MN410627 | *E. spinifera* | 35/F with Subcutaneous abscess in axilla with enlarged cervical lymph nodes | None | FNAC pus from lesion KOH mount and culture positive | Itraconazole | Recovered | Present Study |
| 18 | Odisha | 2018 | 106028 | MN410628 | *E. jeanselmei* | 55/M, cystic swelling on the skin over chest (left side) | Type 2 diabetes mellitus- uncontrolled | FNAC: microscopy showed multiple septate hyphae and culture positive | None | Initial improvement with recurrence of swelling after 2 weeks- repeat drainage performed. LFU | Present Study |
| 19 | Hyderabad | 2019 | 106029 | MN410629 | *E. dermatitidis* | 52/M, intermittent fever and chest pain | Hypertension, aortic valve replacement surgery | Culture of aortic valve positive | Re-operative mechanical valve replacement, intravenous voriconazole | Recovered | Present Study |
| 20 | Chandigarh | 2019 | 106030 | MN410630 | *E. dermatitidis* | 20/M with nasal polyp, allergic fungal rhinosinusitis | None | Nasal tissue : Culture positive | Itraconazole | LFU | Present Study |
| 21 | Pune | 2019 | 106031 | MT649873 | *E. dermatitidis* | 28/M with fever, headache followed by abnormal body movements and impaired consciousness | None | Cerebral MRI revealed multiple lesions and CSF culture positive | Voriconazole | Death | Present Study |
| 22 | Odisha | 2019 | 106032 | MT649874 | *E. jeanselmei* | 37/ M laborer with subcutaneous swelling over right foot | None | Drained pus : Culture positive | Itraconazole | Recovered | Present Study |
| 23 | Jodhpur | 2020 | 106033 | MW724320 | *Exophiala arunalokei s*p. nov*.* | 35/M with Subcutaneous phaeohyphomycosis involving hand and face | None | FNAC culture positive | Surgical excision | LFU | Present Study |

**References**

Kapatia G., Pandey T., Kakkar N., Kaur H., and Verma R. (2018). Facial phaeohyphomycosis in an immunocompetent individual. Am. J. Dermatopathol. 00, 1. doi: 10.1097/DAD.0000000000001261.

Patel A. K., Patel K. K., Darji P., Singh R., Shivaprakash M. R., and Chakrabarti A.(2013). *Exophiala dermatitidis* endocarditis on native aortic valve in a post renal transplant patient and review of literature on *E. Dermatitidis*. Infect. Mycoses. 56, 365–372. doi: 10.1111/myc.12009

Purnima B., Rudramurthy S. M., Devi U., Kakati S., and Ajanta Sharma J. M. (2015). Isolation of *Exophiala mesophila* from respiratory tract of an immunocompromised patient. Int. J. Heal. Sci. Res. 5, 449–453. doi: https://doi.org/10.52403/ijhsr

Singh G., Shivaprakash M. R., De D., Gupta P., Gupta S., Kanwar A. J., et al. (2012). Chronic disfiguring facial lesions in an immunocompetent patient due to *Exophiala spinifera:* a case report and review of literature. Mycopathologia 174, 293–299. doi: 10.1007/s11046-012-9548-5

Sood S., Vaid V. K., Sharma M., and Bhartiya H. (2014). Cerebral Phaeohyphomycosis by *Exophiala dermatitidis.* Indian J. Med. Microbiol. 32, 188–190. doi: 10.4103/0255-0857.129830

Srinivas S. M., Gowda V. K., Mahantesh S., Mannapur R., and Shivappa S. K. (2016). Chromoblastomycosis associated with bone and central nervous involvement system in an immunocompetent child caused by *Exophiala spinifera*. Indian J. Dermatol. 61, 324–328. doi: 10.4103/0019-5154.182425

Venkateshwar S., Ambroise M. M., Asir G. J., Mudhigeti N., Ramdas A., Authy K., et al. (2014). A rare case report of subcutaneous phaeohyphomycotic cyst caused by *Exophiala oligosperma* in an immunocompetent host with literature review. Mycopathologia 178, 117–121. doi: 10.1007/s11046-014-9762-4
