# Supplementary material for: Clinical Spectrum, Molecular Characterization, Antifungal Susceptibility Testing of Exophiala spp. From India and Description of a Novel Exophiala Species, E. arunalokei sp. nov
Source: Front Cell Infect Microbiol. 2021 Jul 2;11:686120. doi: 10.3389/fcimb.2021.686120 (PMC8284318; doi:10.3389/fcimb.2021.686120)
Supplement: Supplementary file 5 [file Table_2.docx]

**Table S2. Details of additional case studies of infections due to *Exophiala* spp reported from India (isolates not included in present study)**

KOH 10% potassium hydroxide mount, NA- not available, M male, F female, FNAC: fine needle aspiration cytology, GMS: Grocotts methanamine silver stain, H and E: hematoxylin and Eosin stain, PAS: periodic acid schiff’s stain, BD twice a day, LFU lost to follow up, USG ultrasonogram, ITS intergenic transcribed spacer region, MF Masson fontana stain

| **Sno** | **Study details** | **Species** | **Clinical detail** | **Risk factor** | **Investigation** | **Treatment** | **Outcome** |
| --- | --- | --- | --- | --- | --- | --- | --- |
| 1 | Thammavya A et al 1980  Chennai | *E. jeanselmei* | 65/M businessman with painless right lower leg and foot swelling, multiple discharging sinuses with black-brown granules | None | X ray: cortical erosion of lower end of tibia, soft tissue swelling and demineralization of metatarsal heads  KOH mount and culture of sinus discharge was positive | NA | NA |
| 2 | Singh SM et al 1992  Jabalpur | *E. jeanselmei* | cutaneous phaeohyphomycosis | NA | NA | NA | NA |
| 3 | Rajendran C et al 2003  Allahabad | *E. spinifera* | 12/F, student, multiple verrucous plaques on face for 1.5 years progressing to involve upper chest, arms and thighs | None | lesional material was excised and for direct KOH microscopy , histopathology (H and E, PAS)and culture were positive  Axillary lymphnode biopsy: positive for fungal elements | Itraconazole 100mg BD orally- 3 months | Recovered |
| 4 | Capoor MR et al, Uttar Pradesh 2007 | *E. jeanselmei* | 8/M  black grain eumycetoma of foot with osteomyelitis | Injury with wooden splinters, malnourished | KOH and culture positive | Ketoconazole +surgical debridement | Recovered |
| 5 | Gabhane et al,  Maharsahtra 2008 | *E. jeanselmei* | 30/M, eumycetoma of foot with bone involvement | None | Cytology (PAS, HE) and culture positive | NA | NA |
| 6 | Singal A et al, 2008 | *E. spinifera* | 10/M | None | KOH revealed dark, thick-walled, globose, dematiaceous, budding yeast/fungal cells in chains., HE stain - budding yeast cells in dermis. Culture was positive | itraconazole+ fluconazole+ cryotherapy🡪 terbinafine | LFU |
| 7 | Radhakrishnan D et al 2010  Chennai | *E. spinifera* | 20/F, multiple non healing skin ulcers over body for 6 months | Malnourished, anaemic, Hepatitis B surface Ag positive | Wedge biopsy of ulcer histopathology revealed black pigmented hyphal elements, KOH mount and culture positive | oral ketoconazole 200 mg/day | Recovered |
| 8 | Sathyabhama et al  2014  Thiruvananthapuram, | *E. jeanselmei* | 42/M, swelling over right foot for 2 years | Diabetic and hypertensive. History of injury with wooden splinter | FNAC from swelling: KOH mount and culture positive | Oral ketoconazole- 2 months | Recovered |
| 9 | Karunakarreddy CH et al, 2014,  Mangalore | *E. dermatitidis* | 52/M, farmer with swelling over planter aspect of right foot | trauma on foot | USG: thick walled multilocular collection with thick echogenic debris extending from the mid foot to the fore foot upto the first and second toes | Surgical excision, Histopathology (PAS) and culture positive | Recovered |
| 10 | Bhardwaj S et al  2016  Delhi | *E. jeanselmei* | 30/F, swelling on lateral aspect of left forearm since 6 months | Cook with burn scar (16 years old) at site of lesion. No other significant history | X-ray elbow joint and forearm: subcutaneous swelling without  extension to the bones. FNAC from swelling-KOH mount and culture positive, Histopathology (PAS) positive. Identified by ITS and DI/D2 region sequencing | Excision of lesion. No antifungal | Recovered |
| 11 | Joshi P et al, 2016  New Delhi | *E. jeanselmei* | 48/M, painless nodular swelling obver right ankle | Renal transplant recipient (6 months ago). He received basiliximab, cortisteroids, tacrolimus, mycophenolate mofetil | FNAC was Histopathology (Papanicolaou and H and E), KOH mount, and culture positive | surgical excision and amphotericin B followed by itraconazole for 6 weeks | Recovered |
| 12 | Chintagunta S et al 2017  Secunderabad | *E. jeanselmei* | 35/M, agriculturist with multiple asymptomatic swellings on dorsum of hand and feet. Discharging sinus with yellow discharge | agriculturist | Cyst material: Histopathology (PAS and GMS) positive and culture positive. | surgical excision, no antifungal | Recovered |
| 13 | Gupta AJ et al,  2017, New Delhi | *E. dermatitidis* | 15/F, left breast lump with enlarged cervical and axillary lymph nodes for 4 months | None | FNAC was histopathology (Giemsa, PAS and GMS) and culture positive | NA | NA |
| 14 | Satish H et al, 2017,  Puducherry | *E. jeanselmei* | 16/M, student with skin nodule on right leg | Renal transplant recipient received basiliximab | Histopathology (PAS, GMS, MF) | Surgical excision, Itraconazole 200 mg/day for 3 months | Recovered |
| 15 | Monika H et al 2018 Coimbatore | *E. dermatitidis* | 59/M , pain and redness in left eye for 15 days | Cataract surgery with intraocular lens implantation in left eye 4 months ago | Indirect ophthalmoscopy: vitritis (Left eye), with fluffy deposits on posterior surface of intraocular lens. Microscopy and Culture of biopsy from posterior capsule was positive. Identified by ITS sequencing (Accession no. LN809939.) | Intravitreal  injections of voriconazole (100 lg/0.1 ml) for 2 weeks followed by topical  voriconazole (1%)-2 months and systemic fluconazole (150 mg twice daily)- 1 month | Recovered |
| 16 | Ramprasad et al 2020, Delhi | *E. jeanselmei* | 30-year-old male, cattle trader with recurrent and progressive uclerative nodular lesions on face trunk and back. Subsequent tibial osteomyelitis and right arm involvement: disseminated phaeohyphomycosis | Possible traumatic inoculation | KOH mount and culture from ultrasound guided aspirate from right arm was positive | amphotericin B and flucytosine 4 weeks of IV treatment followed by Itraconazole (200 mg BID) and terbinafine (250 mg BD) | Recovered |

**References**

Bhardwaj S., Capoor MR., Kolte S., Purohit G., Dawson L., Gupta K., et al. (2016). Phaeohyphomycosis Due *to Exophiala jeanselmei:* An Emerging Pathogen in India—Case Report and Review. Mycopathologia 181, 279–284. doi: 10.1007/s11046-015-9955-5.

Capoor M., Khanna G., Nair D., Hasan A., Rajni., Deb M., et al. (2007). Eumycetoma pedis due to *Exophiala jeanselmei.* Indian J Med Microbiol 25, 155. doi: 10.4103/0255-0857.32726

Chintagunta S., Arakkal G., Damarla S., Vodapalli A. (2017). Subcutaneous phaeohyphomycosis in an immunocompetent Individual: A case report. Indian Dermatol Online J 8, 29. doi: 10.4103/2229-5178.198770.

Gabhane SK., Gangane N., Anshu N. (2008). Cytodiagnosis of Eumycotic Mycetoma. Acta Cytol 52, 354–356. doi: 10.1159/000325522.

Gupta AJ., Singh M., Yadav S., Khurana N., Jain SL., Chawla R., et al. (2017). Phaeohyphomycosis breast masquerading as fibroadenoma in a young teenage girl. Diagn Cytopathol 45, 939–942. doi: 10.1002/dc.23755.

Joshi P., Agarwal S., Singh G., Xess I., Bhowmik D. (2016). “A fine needle aspiration cytology in time saves nine” - cutaneous phaeohyphomycosis caused by *Exophiala jeanselmei* in a renal transplant patient: Diagnosis by fine needle aspiration cytology. J Cytol 33, 55–57. doi: 10.4103/0970-9371.175529.

Karunakarreddy CH., Thejaswids PC., Kini H., Shenoy S., Prabhu S.(2014). Phaeohyphomycotic cyst in the foot by *Exophiala.* J Clin Diagnostic 8, ND10–ND11. doi: [10.7860/JCDR/2014/9495.5184](https://dx.doi.org/10.7860%2FJCDR%2F2014%2F9495.5184)

Homa M., Manikandan P., Saravanan V., Revathi R., Anita R., Narendran V., et al. (2018) *Exophiala dermatitidis* Endophthalmitis: Case Report and Literature Review. Mycopathologia 183, 603–609. doi: 10.1007/s11046-017-0235-4.

Radhakrishnan D., Jayalakshmi G., Madhumathy A., Thasneem Banu S., Geethalakshmi S., Sumathi G. (2010) Subcutaneous phaeohyphomycosis due to *Exophiala spinifera* in an immunocompromised host. Indian J Med Microbiol 28, 396–399. doi: 10.4103/0255-0857.71838.

Rajendran C., Khaitan BK., Mittal R., Ramam N., Bhardwajw K M., Datta K. (2003). Phaeohyphomycosis caused by *Exophiala spinifera* in lndia. Med Mycol 41:437–441. doi: 10.1080/1369378031000153820.

Ramprasad A., Rastogi N., Xess I., Singh G., Ranjan P., Jadon R., et al. (2020) Disseminated phaeohyphomycosis by *Exophiala jeanselmei*. *QJM* , 113:305. doi: 10.1093/qjmed/hcz298.

Satish H., Parameswaran S., Bheemanathi S. H., Chandrasekhar L., Suseela B. B., Singh R., et al. (2017). Subcutaneous Phaeohyphomycosis in Kidney Transplant Recipients: A Series of Seven Cases. Transpl. Infect. Dis. 19, e12788. doi: 10.1111/tid.12788

Singal A., Pandhi D., Bhattacharya SN., Das S., Aggarwal S., Mishra K. et al. (2008) Pheohyphomycosis caused by *Exophiala spinifera*: A rare occurrence. Int J Dermatol 47, 44–47. doi: 10.1111/j.1365-4632.2007.03430.x.

Singh SM., Pouranik M., Naidu J. (1992) Cutaneous Phaehyphomycosis Caused by *Exophiala Jeanselmei* Var Lecanii-Cornii (Benedek and Specht) De Hoog. Indian J Pathol Microbiol 35, 269–73.

Sathyabhama., Bhageerathi S., Raj S. (2014) Mycetoma caused by *Exophiala jeanselmei.* J Acad Clin Microbiol 16, 94. **doi:** 10.4103/0972-1282.144734

Thammayya A., Sanyal M. (1980) *Exophiala jeanselmei* causing mycetoma pedis in India. Med Mycol 1980, 18:91–95.
